# Expression Profile of Genes Encoding Proteins Involved in Regulation of Vasculature Development and Heart Muscle Morphogenesis—A Transcriptomic Approach Based on a Porcine Model

**DOI:** 10.3390/ijms22168794

**Published:** 2021-08-16

**Authors:** Mariusz J. Nawrocki, Karol Jopek, Maciej Zdun, Paul Mozdziak, Marek Jemielity, Bartłomiej Perek, Dorota Bukowska, Bartosz Kempisty

**Affiliations:** 1 Department of Anatomy, Poznan University of Medical Sciences, 60-781 Poznań, Poland; mjnawrocki@ump.edu.pl; 2 Department of Histology and Embryology, Poznan University of Medical Sciences, 60-781 Poznań, Poland; karoljopek@ump.edu.pl; 3 Department of Basic and Preclinical Sciences, Institute of Veterinary Medicine, Nicolaus Copernicus University in Toruń, 87-100 Toruń, Poland; maciejzdun@umk.pl; 4 Physiology Graduate Program, North Carolina State University, Raleigh, NC 27695, USA; pemozdzi@ncsu.edu; 5 Prestage Department of Poultry Science, North Carolina State University, Raleigh, NC 27695, USA; 6 Department of Cardiac Surgery and Transplantology, Poznan University of Medical Sciences, 61-848 Poznań, Poland; kardiock@ump.edu.pl (M.J.); bperek@ump.edu.pl (B.P.); 7 Department of Diagnostics and Clinical Sciences, Institute of Veterinary Medicine, Nicolaus Copernicus University in Toruń, 87-100 Toruń, Poland; dbukowska@umk.pl; 8 Department of Veterinary Surgery, Institute of Veterinary Medicine, Nicolaus Copernicus University in Toruń, 87-100 Toruń, Poland

**Keywords:** coronary vessels, neovascularization, cell culture, transcriptomic analysis

## Abstract

Despite significant advances in treatment of acute coronary syndromes (ACS) many subjects still develop heart failure due to significantly reduced ejection fraction. Currently, there are no commonly available treatment strategies that replace the infarcted/dysfunctional myocardium. Therefore, understanding the mechanisms that control the regeneration of the heart muscle is important. The development of new coronary vessels plays a pivotal role in cardiac regeneration. Employing microarray expression assays and RT-qPCR validation expression pattern of genes in long-term primary cultured cells isolated form the right atrial appendage (RAA) and right atrium (RA) was evaluated. After using DAVID software, it indicated the analysis expression profiles of genes involved in ontological groups such as: “angiogenesis”, “blood vessel morphogenesis”, “circulatory system development”, “regulation of vasculature development”, and “vasculature development” associated with the process of creation new blood vessels. The performed transcriptomic comparative analysis between two different compartments of the heart muscle allowed us to indicate the presence of differences in the expression of key transcripts depending on the cell source. Increases in culture intervals significantly increased expression of SFRP2, PRRX1 genes and some other genes involved in inflammatory process, such as: CCL2, IL6, and ROBO1. Moreover, the right atrial appendage gene encoding lysyl oxidase (LOX) showed much higher expression compared to the pre-cultivation state.

## 1. Introduction

Cardiovascular diseases are the most common cause of death worldwide. Coronary artery disease (CAD), manifested frequently by myocardial infarction and, subsequently, end-stage heart failure (HF), are still an unsolved clinical problems that focus the attention of the scientific world [1]. Unfortunately, young patients constitute a large group of those affected, and they should be the main group of interest in the search to find effective therapeutic options. Effective and fast development of new coronary blood vessels seems to be essential for regeneration of the injured myocardium and eventually restoration of cardiac hemodynamic function [2]. Tian et al. suggest that distinct coronary vessels arise de novo in the postnatal stage that are not from the embryonic coronary vessels [3].

Neovascularization is the process of blood vessel development and growth through three distinct biological processes: vasculogenesis (limited to the embryonic development), angiogenesis, and arteriogenesis [4]. It is now well established that mammalian heart after injury heals primarily by fibrosis. Cardiac fibroblasts, as a major cell population of the heart, proliferate at the site of injury and, by synthesis of the collagen-rich extracellular matrix (ECM) network, play a pivotal role in cardiac fibrosis [5,6]. During the compound process of cardiac repair, cardiac fibroblasts are key players and their proliferation is often accompanied by recruitment of blood vascular endothelial cells [7,8]. Endothelial cells, present at the site of injury and stimulating repair processes, in particular neovascularization, may also generate new fibroblast subsets by undergoing endothelial–mesenchymal transition (EndoMT) [9,10]. This source of fibroblasts forms the basis of the first model to explain stimulation of neovascularization during cardiac regeneration. Ubil et al. indicate that resident fibroblasts generate a substantial number of endothelial cells in the injured heart tissues [11]. It was demonstrated, by employing genetic fate map techniques (based on Col1a2-CreERT transgene), that cardiac fibroblasts rapidly adopt an endothelial-cell-like phenotype through mesenchymal–endothelial transition (MEndoT) in the heart after injury.

However, previous studies demonstrate that resident fibroblast lineages mainly mediate cardiac fibrosis [7,12], suggesting that most fibroblasts adopt fibroblast cell fate after cardiac injury. Thus, it appears preexisting endothelial cells mainly mediate neovascularization after injury, and non-endothelial cells like fibroblasts contribute minimally, if any, to endothelial cells. The fibroblast-associated genetic lineage tracing data, presented by He et al. [13], indicated that fibroblasts do not contribute significantly to endothelial cells in the injured heart, and thus pointing to no contribution of MEndoT to neovascularization. Instead, it was shown that preexisting endothelial cells are essential for creation of new coronary vessels.

The aim of the present study was to analyze the transcriptomic profile of genes encoding proteins that could be recognized as new molecular markers regulating neovascularization process in the cardiac muscle during in vitro long-term primary cell culture. The pivotal role of the vascular network for development and regeneration of cardiac muscle stimulated research aimed at creating a specific transcriptomic map for cultured porcine myocardial cells. The present study focuses on factors related to the process of creating new vessels, and the expression patterns depending on the duration of the cultivation and the heart compartment from which the cells were isolated (right atrial appendage and right atrium) are considered.

## 2. Results

**Figure 1:**
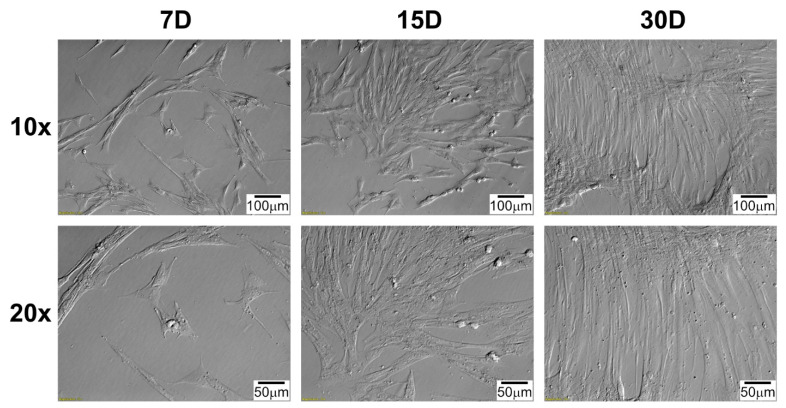
Changes in right atrial appendage (RAA) cells morphology during long-term in vitro primary culture at individual time intervals. D: day of culture; 10×, 20×: magnification.

**Figure 2:**
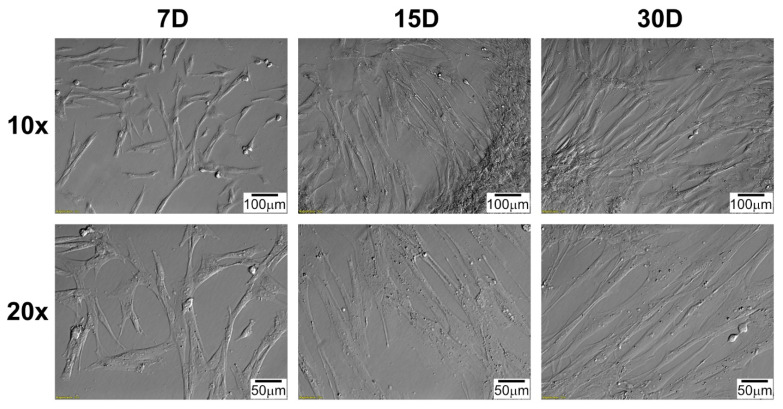
Changes in right atrium (RA) cells morphology during long-term in vitro primary culture at individual time intervals. D: day of culture; 10×, 20×: magnification.

Daily observation of cell cultures, during 30 days of in vitro culture, was performed, and documented with pictures from inverted microscope employing relief contrast (IX73, Olympus, Tokyo, Japan; Figure 1 and Figure 2). The morphology of the cells in the cultures obtained, regardless of the source of the cells, is very similar. Characteristically, first cells appear on the bottle bottom 4–5 days after the culture was established. Cardiac cells changed their shape from irregular, slightly elongated to clearly spindle-like during primary in vitro culture. Observed cells adopted an elongated shape, and form densely covered cell clusters. Typical of cultured cells was the tendency for the cells to overlap with longer cultivation, even in the absence of significant confluence.

**Figure 3:**
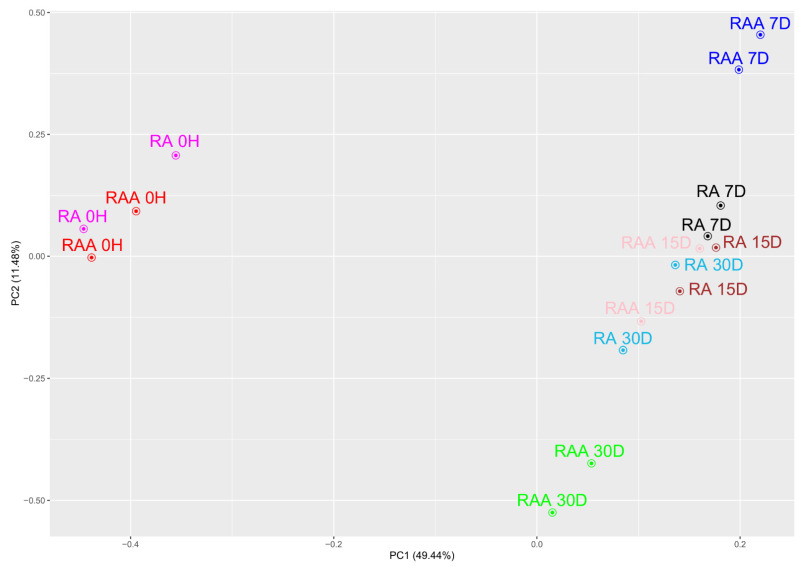
Principal component analysis (PCA) plot of all differentially expressed genes (DEGs), based on the first two principal components (PC1 and PC2) loadings against each other. Percentage of variance is given in brackets.

Changes in the transcriptome profile of cultured cells were observed at individual time intervals. Whole transcriptome profiling by Affymetrix microarray allowed us to analyze gene expression changes between the starting point and 7, 15, and 30 days of pig cardiac cells in vitro primary culture. Using Porcine Gene 1.1 ST Array Strip, microarray transcriptome screening was performed. For both cells obtained from RAA and RA, we observed upregulated and downregulated genes. Genes considered as differentially expressed and selected for downstream analysis showed ratio higher than abs |2| and corrected *p*-values less than 0.05. This set of genes consisted of 4239 differentially expressed genes (DEGs) for RA and 4662 DEGs for RAA. During the initial analysis of the microarray results, to better understand the intergroup differences in the global expression of overall DEGs, principal component analysis (PCA) of the samples was performed to examine variance between the analyzed sample groups. The results presented in Figure 3 clearly indicate the presence of four main clusters: transcript expression levels obtained from cells before cultivation (0H) both for RA and RAA groups create the first one; results for RAA 7D and RAA 30D provide two next clusters, whereas other groups create the last cluster. Overall, there is a major variance between the sample groups representing different culture period for RAA, with less differences among samples for RA, where RA 7D, RA 15D and RA30D groups are focused in one cluster. Additionally, variation within all analyzed groups is negligible, indicating similar gene expression changes evoked by long-term in vitro cultures of individual samples (for example both RAA 7D or RA 15D). However, our present study focuses on genes related to the process of creating new vessels, and we have identified 224 different transcripts for RA and 222 for RAA. The 5 most significantly upregulated and downregulated genes obtained for RAA and RA in all culture periods (7D, 15D, 30D) in relation to the transcript levels before cultivation (0H), their symbols, fold changes and corrected *p*-values are shown in Supplementary Tables S1–S6. In all the attached tables, a common group of genes (17) is noticeable, the expression level of which varies depending on the source of the cells, whereas the direction of expression change (upregulation or downregulation) in both cell cultures was maintained.

**Figure 4:**
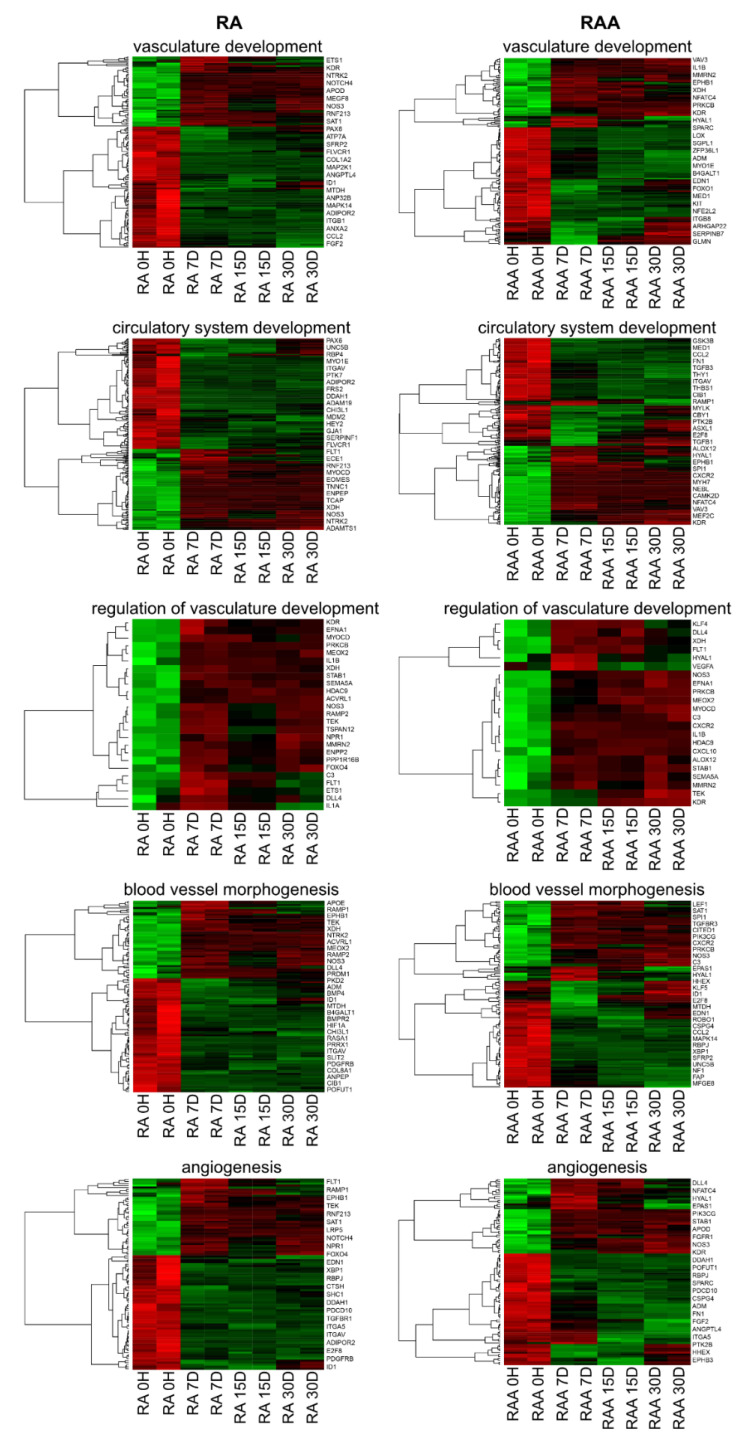
Heatmaps with hierarchical clusterization of the differentially expressed genes, both in right atrial appendage (RAA) and right atrium (RA), belonging to “angiogenesis”, “blood vessel morphogenesis”, “circulatory system development”, “regulation of vasculature development”, and “vasculature development” GO BP terms. Each separate row on the y-axis represents a single transcript. Normalized signal intensity acquired from the microarray analysis is represented by color (green = higher expression; red = lower expression).

The DAVID software analysis showed that the differently expressed genes belonged also to terms associated with the formation of new vessels, like: “angiogenesis”, “blood vessel morphogenesis”, “circulatory system development”, “regulation of vasculature development”, and “vasculature development” GO BP terms. Hierarchical clusterization procedure was carried out for sets of these genes, with the results presented as heatmaps (Figure 4).

**Figure 5:**
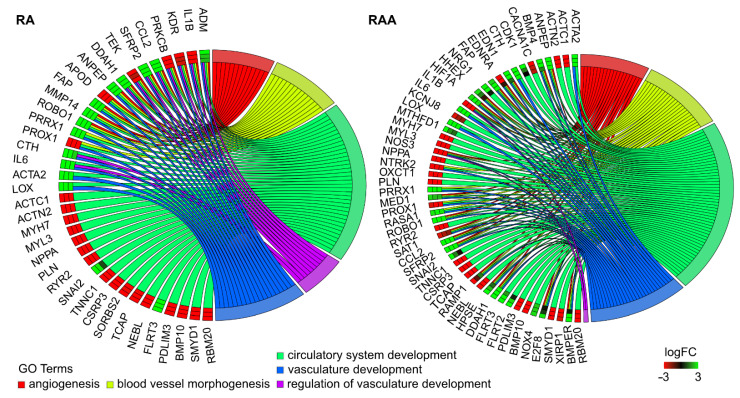
The representation of the mutual relationship between genes that belong to “angiogenesis”, “blood vessel morphogenesis”, “circulatory system development”, “regulation of vasculature development”, and “vasculature development” GO BP terms. The ribbons show the genes belonging to the given categories. The color bars near each gene correspond to logFC between culture intervals.

Due to the structure of the GO database, single genes can often be assigned to many ontological terms. For this reason, we explore the gene intersections between the selected GO BP terms, and the relationship between genes and GO terms were mapped with circle plots, with visualization of logFC values and gene symbols (Figure 5). All of those genes were either upregulated or downregulated in the cells culture intervals compared to controls.

**Figure 6:**
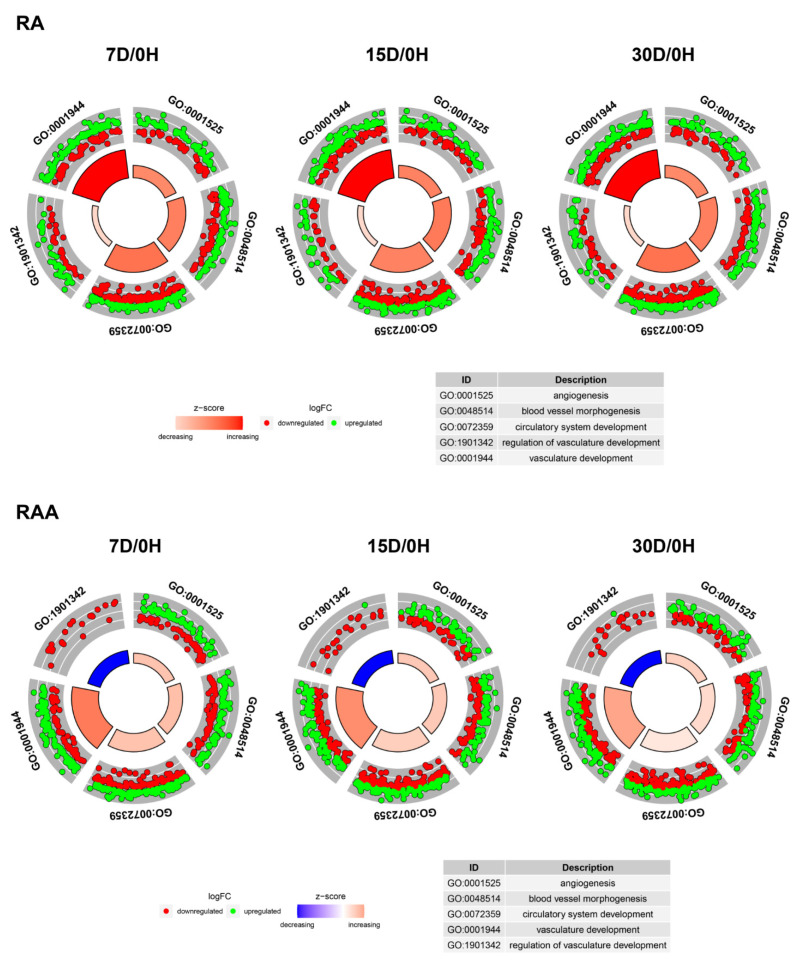
The circle plot showing the differently expressed genes and z-score of the GO BP terms of interest. The outer circle shows a scatter plot for each term of the fold change of the assigned genes. The inner circle shows the z-score of each GO BP term. The width of each bar corresponds to the number of genes within a GO BP term, and the color corresponds to the z-score.

In order to further investigate the changes within chosen GO BP terms, we measured the enrichment levels of each selected GO BP term. The enrichment levels were expressed as z-score and presented as circular visualization (Figure 6).

**Figure 7:**
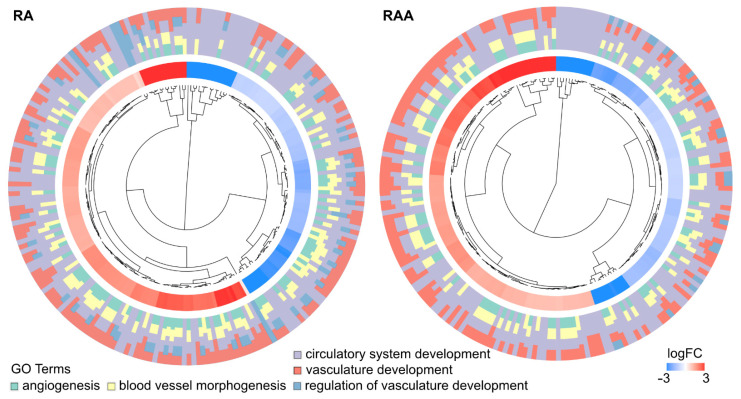
The representation of hierarchical clusterization, FC and assignment of differently expressed genes that belong to “angiogenesis”, “blood vessel morphogenesis”, “circulatory system development”, “regulation of vasculature development”, and “vasculature development” GO BP terms. Genes are grouped together based on their expression patterns, and the clusterization pattern is represented by dendrogram inside the circle. The middle ring represents the logarithm of gene expression FC of studied genes. The outer ring represents the terms assigned to the genes. The genes were clustered based on their logFC values.

Furthermore, to better understand the interaction between chosen GO BP terms, hierarchical clusterization of the gene expression profiles was performed. The dendrogram was combined with fold change (FC) of studied gene expressions and gene assignment to studied terms (Figure 7). All genes belong to the ontologies of interest have been grouped based on their patterns of expression, as well as their mutual associations inside the ontological terms.

**Figure 8:**
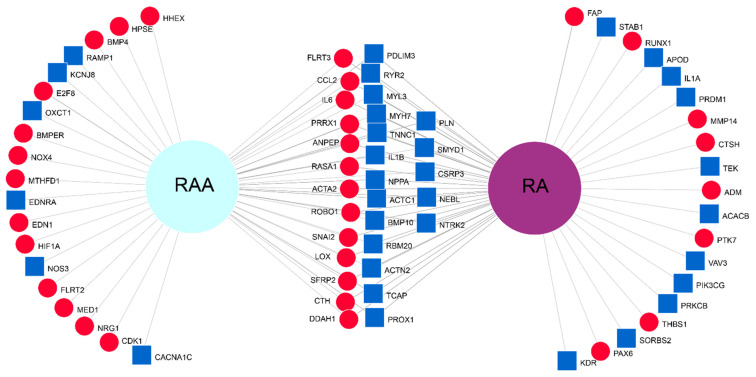
Venn diagram. The 50 most altered genes for RAA and RA were used in the comparison. Blue squares show downregulated genes, whereas red circles—upregulated.

Created Venn diagram, to compare expression profile of two different compartments of the heart, displays the information about the gene expression patterns, namely we can distinguish commonly upregulated and commonly downregulated genes in RA and RAA (Figure 8). As can be seen on the figure, the direction of changes is the same for both analyzed groups. The middle part of the diagram shows common genes in top 50 group both for RA and RAA. In contrast, the gene names listed in the lateral parts of the diagram indicate the DEGs specific only to RAA (left side) and RA (right side of the diagram) in the compared most altered 50 genes group. As can be seen, all the most altered genes listed in the Supplementary Tables S1–S6 and subjected to quantification validation by RT-qPCR are in the center of the diagram.

**Figure 9:**
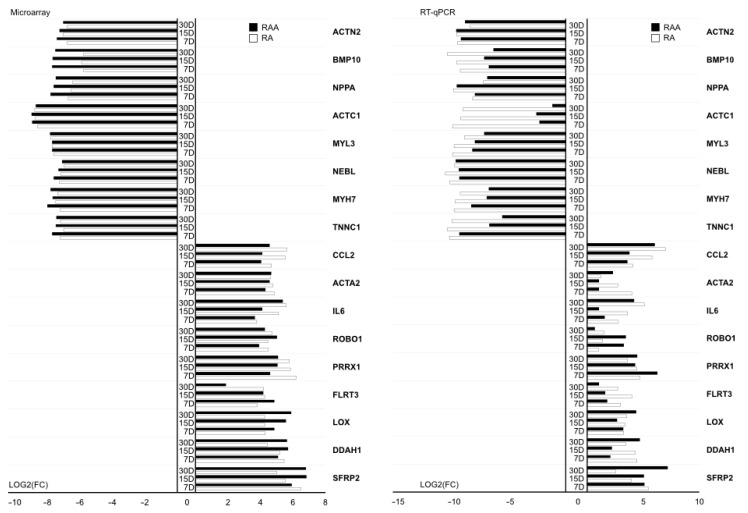
RT-qPCR quantitative validation of microarray results presented in a form of a bar graph. The graph shows the relative changes in gene expression results for three different periods of cell culture (days: 7, 15 and 30) in relation to transcript levels obtained from cells before cultivation. All of the presented sample means were deemed to be statistically significant (*p* < 0.05).

The most significantly upregulated and downregulated genes belonging to GO BP terms of interest, presented on the Venn diagram and in Supplemental Tables S1–S6, were used to validate results obtained during microarray analysis. The RT-qPCR method was applied in order to quantitatively validate the microarray results. The results of RT-qPCR analysis were shown as a bar chart (Figure 9). The left side of the figure shows the results obtained in the microarray analysis, while the right side shows the quantitative validation. Importantly, the direction of change in all 17 genes has been quantified. None of the genes showed any other change in expression than indicated by the results of the expression microarrays. However, the scale of differences in transcript levels varied between both methods analyzed. The most upregulated genes, in RT-qPCR, from the examined DEGs included, among others, SFRP2-secreted frizzled related protein 2, PRRX1-paired related homeobox 1 and CCL2-C–C chemokine ligand 2. The strongest downregulated genes were ACTN2-actinin alpha 2, NEBL-nebulette and, mainly in RA, TNNC1-troponin C type 1 (slow).

**Figure 10:**
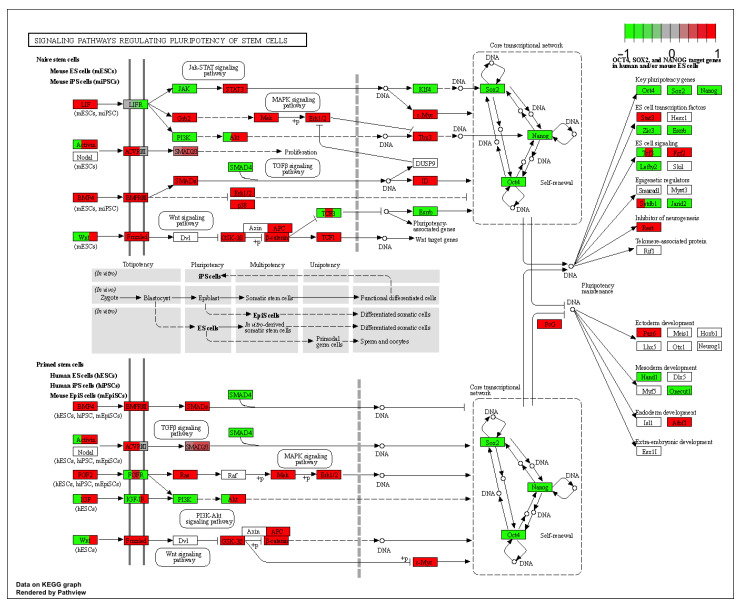
The “signaling pathways regulating pluripotency of stem cells” KEGG pathway analysis of differentially expressed genes. The green and red colors indicate, respectively, upregulated and downregulated gene expression in the relevant comparisons. The boxes with gene names were separated into three parts containing the representation of mRNA expression from the 7th, 15th and 30th day of culture for right atrial appendage (RAA).

**Figure 11:**
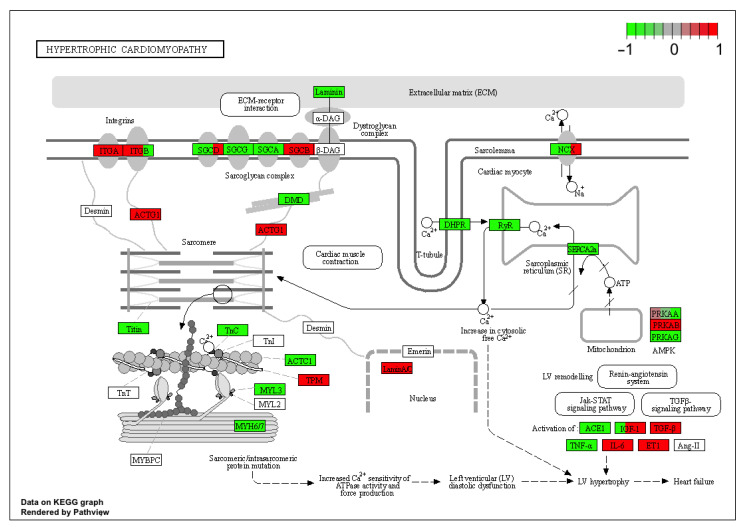
The “hypertrophic cardiomyopathy” KEGG pathway analysis of differentially expressed genes. The green and red colors indicate, respectively, upregulated and downregulated gene expression in the relevant comparisons. The boxes with gene names were separated into three parts containing the representation of mRNA expression from the 7th, 15th and 30th day of culture for right atrial appendage (RAA).

A pathway analysis was also performed for the differentially expressed genes based on the Kyoto Encyclopedia of Genes and Genomes (KEGG) database. This analysis al-lowed us to determine the biological pathways, and for the present study “signaling path-ways regulating pluripotency of stem cells” and “hypertrophic cardiomyopathy” was chosen. Presented involve a significant enrichment of differentially expressed genes in the examined group (*p* < 0.05). Differentially expressed genes belonging to these pathways were assigned to a predetermined color scale, which was subsequently imposed on the gene/protein symbol field (Figure 10 and Figure 11).

## 3. Discussion

A properly functioning vascular network is essential for delivery of oxygen, nutrients and messenger molecules such as hormones and growth factors, as well as removal of carbon dioxide and metabolic products from different tissues. The vascular network is crucial for the functioning of the entire organism, however vascularization seems to play a particularly important role in the heart. The heart is essential for life, but it has limited regenerative capacity in the adult and decreased extent of microvasculature can have serious and irreversible consequences like heart failure (HF). Given these factors, the whole complex process of neovascularization [14] taking place within the heart tissue is so crucial to understanding. Moreover, because the myocardium itself could serve as a source of treatment, via resident cardiac progenitor cells [15] therefore generation of new blood vessels for delivery oxygen and nutrients to newly created cells is necessary for proper cardiac function. Regardless of the source of the cells involved in the development of new coronary vessels, regulation at the molecular level is a key aspect for the effectiveness of vascularization.

Porcine hearts were chosen for this study because easy access to them and more importantly due to many similarities to the human beings. Although significant insight into the molecular and cellular basis has come from small animal models, significant differences exist with regard to cardiovascular characteristics between these models and humans. Therefore, large animal models are essential to develop the discoveries from murine models into clinical therapies and interventions [16]. Moreover, it was shown the porcine heart borne a close resemblance to the human heart in terms of its coronary circulation and hemodynamic similarities and offered ease of implementation of methods and devices from human healthcare facilities [17].

In cells isolated from both the right atrial appendage (RAA) and right atrial (RA) wall during long-term culture both exhibited increased expression of *SFRP2* gene in all analyzed time periods. Secreted frizzled-related proteins (sFRP) are a family of glycoproteins that can bind to Wnt ligands or frizzled (FzD) receptors, thus the SFRP family may be involved in the regulation of Wnt signaling via both the canonical and noncanonical pathways [18]. The Wnt signaling pathway plays important roles in many organ development, including the heart, where it plays a pivotal role in the formation and subsequent expansion of cardiac progenitor cells in the mesoderm [19]. Moreover, both the β-catenin-dependent and β-catenin independent signaling pathways are implicated in angiogenesis in a variety of organs in both normal and pathological conditions [20,21]. Given these facts, Wnt signaling regulation, e.g., by sFRP family, seems to be extremely important in the process of creating new blood vessels. Among the whole group, sFRP2 is considered to be the most potent [22]. Secreted frizzled-related proteins were initially described to be antagonists of Wnt signaling, by sequestration of soluble Wnt ligands, which prevents their binding to FzD receptors [23,24,25]. However, recent studies showed that sFRP have a more complex relationship with the Wnt pathway. Many researchers have proposed an additional agonistic effect on Wnt signaling by direct binding to FzD receptors or by influencing the Wnt activating effect of soluble Wnt ligands. Skah et al. employing sFRP2−/− mice have shown that the antagonistic or agonistic effect of SFRP2 might depend on the expression level [26]. Additionally, further studies provided by Xavier et al. confirmed the hypothesis that sFRP can either promote or suppress Wnt/β-catenin signaling, depending on its concentration and the cellular context [27]. Other investigators proved, that exposure to sFRP1 activated the non-canonical Wnt signaling pathways enhancing the velocity of endothelial cell spreading on laminin and collagen, and finally a pro-angiogenic response was observed [28]. Courtwright et al., employing a chick chorioallantoic membrane (CAM) assay, were the first to discovered a pro-angiogenic effect of sFRP2 [29]. Moreover, authors indicated that sFRP2 exerts pro-angiogenic effects through activation of non-canonical Wnt/Ca2+ pathways, without affecting the canonical Wnt pathway. Furthermore, the upregulation of sFRP2 in the tumor vasculature indicate that this stimulator can exert an angiogenic effect in a wide variety of human tumors [30,31,32]. Other research found that sFRP2 an important mechanism mediating ischemic cardio protection through induction angiogenesis/arteriogenesis [33].

It is difficult to clearly determine the effect of sFRP2 on the Wnt pathway and how it will translate into the formation of new vessels in the heart. Nevertheless, numerous research results suggest a pro-angiogenic effect of sFRP2 overexpression, which may suggest that in the conditions of in vitro culture, the increasing mRNA level of the SFRP2 gene will be a factor promoting the angiogenesis process.

A significant increase in the mRNA levels of paired related homeobox 1 (PRRX1), a member of the paired homeobox family, and functions as a transcription co-activator [34]. PRRX1 is the epithelial-mesenchymal transition (EMT) inducer involved in the organogenesis of many tissues during developmental processes, additionally EMT is associated with dissemination steps in the processes of cancer growth that enables carcinoma cells to lose epithelial properties, gain invasive capacity and acquire stem cell properties [35]. Moreover, expression of PRRX1 is stable in different tissues in the human body, including heart [36]. The role of this transcription factor in creation of new vessels was demonstrated in Ihida-Stansbury et al.’s study, where authors using Prrx1−/− showed the essential role of PRRX1 for the development and integrity of healthy lung blood vessels [37], whereas other investigators described a correlation between PRRX1 expression and angiogenesis in non-small cell lung cancer (NSCLC), and explored this factor as marker of tumor angiogenesis [38]. Higuchi et al. have shown a link between vasculogenesis during rat embryonic pituitary development and the presence of PRRX1-positive mesenchymal stem/progenitor cells [39]. According with results obtained by Wang et al. PRRX1 seems to be promising biomarker in clear cell renal cell carcinoma as an enhancer of new vessels creation [40].

It is well established, that inflammation and angiogenesis are interdependent and highly linked processes in ischemia and tumor formation. A significant upregulation of transcript level of few factors involved in inflammatory process appears to be important. Secretion of C–C chemokine ligand 2 (CCL2) results in the attraction of blood monocytes into sites of inflammatory responses and tumors [41]. This main activity of the member of the CC-chemokine group (characterized by the presence at the N-terminal two cysteine residues adjacent to each other), makes CCL2 an important component involved in wound healing promotion, also by regulation of angiogenesis [42]. Bonapace et al. demonstrated, that anti-CCL2 treatment decreased breast cancer metastases in mice, but interruption of anti-CCL2 treatment precipitated an unexpected influx of monocytes into the metastatic site and overshooting IL-6 levels within the metastatic microenvironment. This led to local enhancement of angiogenesis, metastatic disease and a fatal outcome [43]. CCL2 expression with high expression of IL-6 and subsequent induction of VEGF-A in monocytes and, results in increased local vascular density. Upregulation of mRNA levels of IL6 in both RAA (30D/0H) and RA (15D/0H and 30D/0H) appear to be important. Accumulating evidence establishes IL-6 as a key player in supporting angiogenesis, where by inflammation may help drive tumor formation, growth, and metastasis [44]. Some studies have also shown, that IL6 signaling can lead to STAT3 activation, further by promoting expression of vascular endothelial growth factor (VEGF) and fibroblast growth factor (bFGF) by tumor cells, supporting the rapid vascularization within tumor tissues [45,46,47]. The current transcriptomic screening studies show increased mRNA levels of ROBO1. Roundabout receptors (ROBO) belong to the immunoglobulin (Ig) superfamily of cell adhesion molecules (CAMs) [48]. ROBO together with their Slit ligands form one of the most crucial ligand-receptor pairings among the axon guidance molecules. Rama et al. by creation of conditional knockout mice deficient in various Slit and Robo proteins have found that Slit2 potently and selectively promoted angiogenesis via Robo1 and Robo2 in mouse postnatal retina and in a model of ocular neovascular disease [49]. Other investigators on the model of hepatocellular carcinoma (HCC) demonstrated, that Robo1 expression promoted tumor angiogenesis and may be considered as an alternative target for anti-angiogenesis treatment [50]. After 7 days (7D/0H) of culturing the cells obtained from the RAA, increase transcript expression of the transmembrane protein, which can interact with the intracellular domain of ROBO1, namely FLRT3. The fibronectin leucine rich transmembrane protein (FLRT) family may function in cell adhesion and/or receptor signaling [51]. Jauhiainen et al. described the role of FLRT3 in the regulation of angiogenesis and vascular patterning via modulation the VEGF-signaling [52]. Moreover, gene-targeting experiments in mice demonstrate demonstrated that Flrt2 is required in the epicardium to promote heart morphogenesis [53].

High transcript levels were observed in some genes, but significantly lower mRNA levels for many genes coding mainly structural proteins, such as like myosin (*MYH7*, *MYL3*), actin (*ACTC1*), actinin (*ACTN2*) or troponin (*TNNC1*) were observed. The expression profile at the molecular level appears to promote blood vessel formation when we look at factors positively regulating neovascularization (upregulation of *SFRP2, PRRX1* or cytokines). However, in the case of genes encoding structural proteins, there is a noticeable need to further optimize the culture conditions, because without specific “building blocks” that build vessels, the expression of transcription factors alone that stimulate vascularization will be insufficient for the formation of new vessels.

## 4. Materials and Methods

### 4.1. Animals

Porcine (*Sus scrofa f. domestica*) hearts, delivered on ice in the shortest possible time after slaughter, from a local slaughterhouse were the source of cells for in vitro culture. For our study, a pubertal crossbred Polish Landrace (PBZ × WBP) gilts, bred on commercial local farm were used. They had a mean age of 155 days (range 140–170 days) and the mean weight were 100 kg (95–120 kg). All the animals were housed under identical conditions and fed the same forage.

### 4.2. Tissue Collection from Porcine Hearts

The hearts were excised within 25 min of slaughter. The heart, located in the pericardium, is situated in the middle mediastinum on the ventral side, between the 3rd and 6th rib. A slightly larger portion is located on the left side of the body (60%). The base of the heart is directed dorsally and cranially while the apex of the heart is directed ventrally, caudally and slightly to the left. In this position, the heart is held in place by the cranial vena cava, the caudal vena cava, the aorta and the sternopericardial ligaments. After cutting the sternum and the diaphragm, the heart was removed along with the lungs, trachea, esophagus and tongue. The hearts in pericardium were then severed and transported to the laboratory within 30 min. Each time the delivered hearts will be assessed for their suitability for downstream analyzes. Hearts were disqualified from further study by observations of macroscopic injury at the slaughterhouse; contaminated during transport and preliminary preparation; with evident signs of inflammation (connective tissue adhesions) or ischemia (patchy necrosis, thinning of any segment of the ventricular myocardium). Then, the hearts were removed from the pericardial sacs. After identification of left and right side of porcine heart, right atrial appendage and piece of its free wall (ca. 1 cm × 1 cm) of whole thickness were extracted. The extracted fragments were manually prepared with surgical instruments to remove the visceral lamina of the serous pericardium (epicardium). As the research material is usually disposed of after slaughter, being a remnant by-product, no ethical committee approval is needed for the project.

### 4.3. Enzymatic Dissociation and Primary Cell Culture

The right atrial appendage (right auricle) and right atrium were extracted from the delivered material, washed in ice-cold PBS solution, to remove the blood and, in the next stage, after the two-step mincing by sterilized scissors in Petri dishes, the tissue undergoes enzymatic digestion in DMEM + collagenase type II (2 mg/mL) solution conducted in 37 °C for 40 min with gentle mixing. After the end of digestion, the remaining tissue will be separated with nylon strainers of 70 μm pore size. The filtrate (containing cells of interest) was centrifuged (5 min, 200× *g*, RT), in order to remove the remaining collagenase from the cell environment. Cell pellet obtained was washed with the PBS buffer and then initially placed on 25 mL cultures bottles in culture medium (DMEM/F12, Sigma-Aldrich, Saint Louis, MO, USA), 20% FBS (Foetal Bovine Serum, Gibco, Thermo-Fischer Scientific, Waltham, MA, USA), 10% HS (Horse Serum, Gibco, Thermo-Fischer Scientific, Waltham, MA, USA), EGF (20 ng/mL; Sigma-Aldrich, Saint Louis, MO, USA), bFGF (10 ng/mL; Sigma-Aldrich, Saint Louis, MO, USA) 1% P/S, and preincubated for 4 h in 37 °C, 5% CO_2_. This stage aims to deplete the fibroblasts, which show much higher adhesion affinity. After this time, the supernatant (including nonadherent cardiac muscle cells) was pelleted by centrifugation (5 min, 200× *g*, RT) and transferred to the new 25 mL culture bottle, previously coated with 0.1% gelatin solution. The cells were cultured in DMEM/F12 complemented with 20% FBS, 10% HS, EGF (20 ng/mL), LIF (10 ng/mL) and 1% P/S at 37 °C in a humidified atmosphere of 5% CO_2_. The culture medium was changed every three days.

### 4.4. Morphological Observation of Cells during Long-Term Primary In Vitro Culture

Using inverted light microscope with relief contrast (IX73, Olympus, Tokyo, Japan) daily observation of cultured cells was performed. In present study, the images obtained during the cultivation period corresponding to the time intervals used in the molecular analysis are shown (7D, 15D, and 30D).

### 4.5. RNA Extraction and Reverse Transcription

Total RNA from all of the samples (both before and after in vitro cultivation) was isolated according to the method published by Chomczyński and Sacchi [54] employing TRI reagent (Sigma-Aldrich; Merck KGaA, Saint Louis, MO, USA). By using successively chloroform, 2-propanol and 75% ethanol during the procedure, we obtained RNA. The RNA samples were re-suspended in 20–40 µL of RNase-free water and stored at −80 °C. RNA integrity was determined by denaturing agarose gel (2%) electrophoresis, and then, the RNA was quantified by measuring the optical density (OD) at 260 nm (NanoDrop spectrophotometer; Thermo Scientific, Inc., Waltham, MA, USA). RNA samples were reverse-transcribed into cDNA using RT2 First Strand kit (Qiagen, Hilden, Germany), according to the manufacturer’s protocol. Then, 500 ng of an RNA sample was used for reverse transcription.

### 4.6. Microarray Expression Study and Data Analysis

In the first step, total RNA (50 ng) from each pooled sample (for all individual time intervals using during analyzes we pooled RNA from 4 different cultures obtained from other hearts) was subjected to two rounds of sense cDNA amplification (Ambion^®^ WT Expression Kit). The synthesis of cRNA was performed by in vitro transcription (16 h, 40 °C). Then, cRNA was purified and re-transcribed into cDNA. Subsequently, cDNA samples were used for biotin labelling and fragmentation using Affymetrix GeneChip^®^ WT Terminal Labeling and Hybridization kit (Affymetrix, Santa Clara, CA, USA). Next, the biotin-labelled samples were loaded onto and hybridized to the Affymetrix^®^ Porcine Gene 1.1 ST Array Strip. Hybridization was conducted at 48 °C for 20 h, in an AccuBlock™ Digital Dry Bath (Labnet International, Inc. NY, USA) hybridization oven. Microarrays were then washed and stained, according to technical protocol, using an Affymetrix GeneAtlas™ Fluidics Station (Affymetrix, Santa Clara, CA, USA). The strips were scanned using an Affymetrix GeneAtlas™ Imaging Station (Affymetrix, Santa Clara, CA, USA). The scans of the microarrays were saved on hard drives as *.CEL files for downstream data analysis.

Quality control (QC) studies were performed using the Affymetrix GeneAtlas™ (Affymetrix, Santa Clara, CA, USA) software, according to the manufacturer’s standards. The generated *.CEL files were subjected to further analysis performed using the R statistical language and Bioconductor package with the relevant Bioconductor libraries. To correct background, normalize and summarize results, we used the Robust Multiarray Averaging (RMA) algorithm. Assigned biological annotations were obtained from the “pd.porgene.1.1.st” library, employed for the mapping of normalized gene expression values with their symbols, gene names, and Entrez IDs, allowing us to generate a complex gene data table. To determine the statistical significance of the analyzed genes, moderated t-statistics from the empirical Bayes method were performed. The obtained *p*-values were corrected for multiple comparisons using Benjamini and Hochberg’s false discovery rate and described as adjusted *p*–values. The selection of significantly altered genes was based on a *p*-value beneath 0.05 and expression higher than two-fold. The differentially expressed gene list (separated for up and down-regulated genes) was uploaded to the DAVID software (Database for Annotation, Visualization and Integrated Discovery). To retrieve gene ontology biological process (GO BP) terms containing differently expressed transcripts, the DAVID software was employed. The selection of significantly altered GO Term was based on a *p*-value (Benajamini) <0.05 and volume of at least 5 genes.

It is important to compare the expression profile in RA and RAA to understand the molecular basis for new vessel formation. A Venn diagram that was used to detect relations between lists of differentially expressed genes in both heart’s compartments and to explore the intersection of genes of analyzed terms from the functional analysis. In order to create the diagram, the 50 most altered genes for both heart compartments were analyzed.

### 4.7. Real-Time Quantitative Polymerase Chain Reaction (RT-qPCR) Analysis

Determination of the transcript levels for analyzed genes was conducted using a Light Cycler^®^ 96 Real-Time PCR System, Roche Diagnostics GmbH (Mannheim, Germany) with SYBR Green as a detection dye. Levels of analyzed transcripts were standardized in each sample, in reference to hypoxanthine 1 phosphoribosyltransferase (HPRT1) and β-actin (ACTB) as an internal control. For each of the amplification reactions, 1 μL of cDNA solution was mixed with 5 μL of mastermix (RT² SYBR Green FAST Mastermix, Qiagen), 3 μL of PCR-grade water and 1 μL of specific starter pair (10 μM). We have used the Primer3 software for primer design (Table 1). The exon–exon design method was used as an additional method to avoid the possible amplification of genomic DNA fragments. The primers were also designed using the sequence of several transcript variants of genes of interest available in the Ensembl database. For target cDNA quantification, we have performed relative quantification with the 2^−ΔΔCq^ method. In order to confirm the specificity of the results and size of amplified products, 2% agarose gel electrophoresis of the products was performed.

## 5. Conclusions

The study evaluated cardiac muscle cells derived from the right atrial appendage and right atrium. The salient of the study was that in vitro condition significantly altered mRNA expression levels in evaluated cells, suggesting that the employed culture conditions may create a favorable environment for neovascularization. The expression profile of factors positively regulating neovascularization such as, SFRP2, PRRX1 or cytokines were upregulated, and ACTN2, ACTC1 or MYH7 were down regulated. Nevertheless, further optimalization of the culture conditions is still needed, because transcript levels of genes coding pivotal structural proteins were significantly lower.

## Figures and Tables

**Table 1:** Primers. Oligonucleotide sequences of primers used for RT-qPCR analysis.

Gene	Primer Sequence (5′–3′)		Product Size (bp)
*SFRP2*	F	GGCCTCAGGAATGGATAGCT	167
	R	CCCCAAACATCACACCCAAG	
*DDAH1*	F	AGCGCGAAGGTATACGAGAA	238
	R	GAAGCGATTAGACTTGGCGG	
*LOX*	F	GTACAACCTGAGATGCGCTG	208
	R	GCTGAATTCGTCCATGCTGT	
*FLRT3*	F	TCGCAACAATCCCTGGTACT	216
	R	ACTGTGTTGGGGATCGAAGT	
*PRRX1*	F	GGACACACTACCCAGATGCT	155
	R	TTTGAGGAGGGAAGCGTTCT	
*ROBO1*	F	GATGTGATTGCAGACCGACC	222
	R	AGTGTCACCCAGCTTAGCAT	
*IL6*	F	ACCGGTCTTGTGGAGTTTCA	170
	R	GCATTTGTGGTGGGGTTAGG	
*ACTA2*	F	CCGAGATCTCACCGACTACC	178
	R	CTCGTAGCTCTTCTCCAGGG	
*CCL2*	F	CCACACCGAAGCTTGAATCC	206
	R	CTTGCTGCTGGTGACTCTTC	
*TNNC1*	F	GAGCTGGGCAAAGTGATGAG	193
	R	ACATGCGGAAGAGGTCAGAA	
*MYH7*	F	CCAACACCAACCTGTCCAAG	173
	R	CAGGATGGGGCAGATCAAGA	
*NEBL*	F	GCACGATCCAGTTTCAGGTC	163
	R	GGCGTTGTCTTTATGGTGCA	
*MYL3*	F	TCTTCGACAAGGAGGGCAAT	191
	R	TTTCCTGGGGTGAGAGGTTC	
*ACTC1*	F	GTCATGGTGGGTATGGGTCA	151
	R	CGTTGTAGAAGGTGTGGTGC	
*NPPA*	F	CAGCAGCCTCTATCCTCTCC	153
	R	CCTGTATCCCTGGCAGTTCT	
*BMP10*	F	CCTGGGTCTGGGTGGTTATT	181
	R	TGGGGCAATGATCCAAGAGT	
*ACTN2*	F	TCGGGGCTGAAGAGATTGTT	189
	R	AGCTGGTGTGGAAGTTCTGA	

## Data Availability

All of the data discussed in this work, if not already included in the manuscript, are available from the corresponding author on reasonable request.

## Supplementary Materials

The following are available online at https://www.mdpi.com/article/10.3390/ijms22168794/s1.
