# Supplementary material for: Expression Profile of Genes Encoding Proteins Involved in Regulation of Vasculature Development and Heart Muscle Morphogenesis—A Transcriptomic Approach Based on a Porcine Model
Source: Int J Mol Sci. 2021 Aug 16;22(16):8794. doi: 10.3390/ijms22168794 (PMC8395751; doi:10.3390/ijms22168794)
Supplement: Supplementary file 1 [file ijms-22-08794-s001.zip › ijms-1293755-supplementary.pdf]

International Journal of
Molecular Sciences

MDPI

Supplementary materials

Table S1. Gene symbols of 10 genes with the largest change expression for RA in 7th day of culture. Fold change in expression ratio, and corrected *p*-values of studied genes.

7D/0H

Gene symbol	Gene name	Ratio	Adj. p.val
SFRP2	secreted frizzled−related protein 2	91.25	<0.05
PRRX1	paired related homeobox 1	73.53	<0.05
DDAH1	dimethylarginine dimethylaminohydrolase 1	43.57	<0.05
ACTA2	actin, alpha 2	28.73	<0.05
CCL2	chemokine (C−C motif) ligand 2	25.94	<0.05
TNNC1	troponin C type 1	−147.83	<0.05
MYH7	myosin, heavy chain 7	−148.79	<0.05
NEBL	nebulette	−151.20	<0.05
MYL3	myosin, light chain 3	−194.43	<0.05
ACTC1	actin, alpha 1	−393.86	<0.05

Table S2. Gene symbols of 10 genes with the largest change expression for RA in 15th day of culture. Fold change in expression ratio, and corrected *p*-values of studied genes.

15D/0H

Gene symbol	Gene name	Ratio	Adj. p.val
PRRX1	paired related homeobox 1	58.05	<0.05
SFRP2	secreted frizzled−related protein 2	45.92	<0.05
CCL2	chemokine (C−C motif) ligand 2	45.80	<0.05
DDAH1	dimethylarginine dimethylaminohydrolase 1	37.06	<0.05
IL6	interleukin 6	34.39	<0.05
ACTN2	actinin, alpha 2	−129.43	<0.05
NEBL	nebulette	−141.50	<0.05
MYH7	myosin, heavy chain 7	−178.28	<0.05
MYL3	myosin, light chain 3	−206.79	<0.05
ACTC1	actin, alpha 1	−422.99	<0.05

Table S3. Gene symbols of 10 genes with the largest change expression for RA in 30th day of culture. Fold change in expression ratio, and corrected *p*-values of studied genes.

30D/0H

Gene symbol	Gene name	Ratio	Adj. p.val
PRRX1	paired related homeobox 1	55.31	<0.05
CCL2	chemokine (C−C motif) ligand 2	49.71	<0.05
IL6	interleukin 6	48.55	<0.05
SFRP2	secreted frizzled−related protein 2	32.02	<0.05
ROBO1	roundabout, axon guidance receptor, homolog 1	26.25	<0.05
NEBL	nebulette	−124.15	<0.05
TNNC1	troponin C type 1	−145.36	<0.05
MYH7	myosin, heavy chain 7	−161.04	<0.05
MYL3	myosin, light chain 3	−219.07	<0.05
ACTC1	actin, alpha 1	−434.00	<0.05

Table S4. Gene symbols of 10 genes with the largest change expression for RAA in 7th day of culture. Fold change in expression ratio, and corrected *p*-values of studied genes.

7D/0H

Gene symbol	Gene name	Ratio	Adj. p.val
SFRP2	secreted frizzled−related protein 2	61.20	<0.05
DDAH1	dimethylarginine dimethylaminohydrolase 1	33.90	<0.05
LOX	lysyl oxidase	29.19	<0.05
FLRT3	fibronectin leucine rich transmembrane protein 3	28.99	<0.05
PRRX1	paired related homeobox 1	24.28	<0.05
MYL3	myosin, light chain 3	−210.94	<0.05
TNNC1	troponin C type 1	−211.77	<0.05
NPPA	natriuretic peptide A	−222.81	<0.05
MYH7	myosin, heavy chain 7	−256.41	<0.05
ACTC1	actin, alpha 1	−490.06	<0.05

Table S5. Gene symbols of 10 genes with the largest change expression for RAA in 15th day of culture. Fold change in expression ratio, and corrected *p*-values of studied genes.

15D/0H

Gene symbol	Gene name	Ratio	Adj. p.val
SFRP2	secreted frizzled−related protein 2	114.50	<0.05
DDAH1	dimethylarginine dimethylaminohydrolase 1	52.44	<0.05
LOX	lysyl oxidase	47.26	<0.05
PRRX1	paired related homeobox 1	33.32	<0.05
ROBO1	roundabout, axon guidance receptor, homolog 1	32.19	<0.05
NPPA	natriuretic peptide A	−196.25	<0.05
MYH7	myosin, heavy chain 7	−205.85	<0.05
BMP10	bone morphogenetic protein 10	−207.29	<0.05
MYL3	myosin, light chain 3	−209.39	<0.05
ACTC1	actin, alpha 1	−510.00	<0.05

Table S6. Gene symbols of 10 genes with the largest change expression for RAA in 30th day of culture. Fold change in expression ratio, and corrected *p*-values of studied genes.

30D/0H

Gene symbol	Gene name	Ratio	Adj. p.val
SFRP2	secreted frizzled−related protein 2	110.94	<0.05
LOX	lysyl oxidase	59.40	<0.05
DDAH1	dimethylarginine dimethylaminohydrolase 1	50.35	<0.05
IL6	interleukin 6	41.71	<0.05
PRRX1	paired related homeobox 1	34.21	<0.05
NPPA	natriuretic peptide A	−180.49	<0.05
BMP10	bone morphogenetic protein 10	−182.54	<0.05
MYH7	myosin, heavy chain 7	−223.37	<0.05
MYL3	myosin, light chain 3	−231.93	<0.05
ACTC1	actin, alpha 1	−423.15	<0.05
